# Flubendiamide Enhances Adipogenesis and Inhibits AMPKα in 3T3-L1 Adipocytes

**DOI:** 10.3390/molecules23112950

**Published:** 2018-11-12

**Authors:** Quancai Sun, Jie Lin, Yukui Peng, Ruichang Gao, Ye Peng

**Affiliations:** 1 School of Food and Biological Engineering, Jiangsu University, Zhenjiang 212001, China; sqctp@hotmail.com (Q.S.); m18352863315@163.com (J.L.); xiyuan2008@ujs.edu.cn (R.G.); 2 Center for Food Quality Supervision & Testing, Ministry of Agriculture, College of Food Science & Engineering, Northwest A&F University, Yangling 712100, China; pyk2009@163.com

**Keywords:** food chemical contaminant, flubendiamide, ryanoid, adipogenesis, AMPKα

## Abstract

Flubendiamide, a ryanoid class insecticide, is widely used in agriculture. Several insecticides have been reported to promote adipogenesis. However, the potential influence of flubendiamide on adipogenesis is largely unknown. The current study was therefore to determine the effects of flubendiamide on adipogenesis utilizing the 3T3-L1 adipocytes model. Flubendiamide treatment not only enhanced triglyceride content in 3T3-L1 adipocytes, but also increased the expression of cytosine-cytosine-adenosine-adenosine-thymidine (CCAAT)/enhancer-binding protein α and peroxisome proliferator-activated receptor gamma-γ, two important regulators of adipocyte differentiation. Moreover, the expression of the most important regulator of lipogenesis, acetyl coenzyme A carboxylase, was also increased after flubendiamide treatment. Further study revealed that 5-aminoimidazole-4-carboxamide ribonucleotide (AICAR) or A769662, two Adenosine 5′-monophosphate (AMP)-activated protein kinase α activators, subverted effects of flubendiamide on enhanced adipogenesis. Together, these results suggest that flubendiamide promotes adipogenesis via an AMPKα-mediated pathway.

## 1. Introduction

The incidence of overweight and obesity has increased dramatically in recent decades and become one of the leading health problems all over the world [1]. Lifestyle and dietary changes alone cannot account for the dramatic rise of overweight and obesity worldwide [2]. An increasing amount of literature shows relationships between exposure to insecticides and obesity in humans and animals [3,4].

Flubendiamide (C23H22F7IN2O4S, CAS No: 272451-65-7), a recently developed phthalic acid diamide insecticide, is very effective and widely used against lepidopterans on over 200 crops. These include corn, fruiting vegetables, grape, cotton, okra, stone fruit, and tobacco. By stimulating ryanodine receptors (ryanodine-sensitive calcium-release channels), flubendiamide disrupts the muscle functions in insects [5]. The wide use of this compound raised the concern about their impact on the environment, food safety and human health. In 2016, the United States Environmental Protection Agency (EPA) issued a Notice of Intent to Cancel all remaining flubendiamide products, due to the continued use of flubendiamide might result in unreasonable adverse effects on the environment, particularly benthic invertebrates [6]. However, this compound is still widely used in some other countries [7]. Therefore, it is of critical importance to better understand the impacts of this compound on the environment, food safety and human health.

Flubendiamide-benzyl alcohol and flubendiamide-benzoic acid were the two major metabolites of flubendiamide in eggs, liver and fat of laying hens. The levels of flubendiamide or its metabolites in fat tissues were approximately 12–14 times higher compared to the levels in muscle tissues in lactating goats [8], suggesting the bioaccumulation of flubendiamide in adipose tissue. The bioaccumulation of this compound in adipose tissue might impair the differentiation or normal function of adipocytes. Previous publications reported the relationships between several food chemical contaminants exposure and obesity, including our previous reports of several types of insecticides promoting adipogenesis in adipocytes [2,9,10,11]. However, the relationship between flubendiamide and adipogenesis is unknown.

Differentiation of 3T3-L1 preadipocytes into adipocytes in cell culture is a good model of the differentiation process in vivo [12]. When treated with an appropriate differentiation protocol, 3T3-L1 preadipocytes lose their fibroblastic features, round-up, and acquire the morphological and biochemical phenotype of adipocytes. In addition, the 3T3-L1 preadipocytes accumulate triglyceride and de novo fatty acid and trigger triglyceride biosynthesis [12]. The current study is therefore to determine the role of flubendiamide in adipogenesis using the 3T3-L1 adipocyte model.

## 2. Materials and Methods

### 2.1. Materials

The 3T3-L1 preadipocytes were from Peking union cell resource center (Beijing, China). Fetal bovine serum (FBS), Dulbecco’s modified Eagle’s medium (DMEM), methylisobutylxanthin, insulin, dexamethasone, dimethyl sulfoxide (DMSO), and 5-aminoimidazole-4-carboxamide ribonucleotide (AICAR) were purchased from Sigma-Aldrich Co. (St. Louis, MO, USA). A769662 was from Tocris Bioscience (Bristol, UK). The contents of triglyceride and protein were quantified with kits from Thermo Scientifics (Middletown, NY, USA) and Bio-Rad Co. (Hercules, CA, USA), respectively. Radioimmunoprecipitation assay (RIPA) buffer supplemented with protease and phosphatase inhibitor was purchased from Beyotime Biotechnology (Shanghai, China). Trizol was obtained from Thermo Scientific (Rockford, AL, USA).

### 2.2. 3T3-L1 Culture

The 3T3-L1 preadipocytes were maintained in DMEM with 10% FBS at 37 °C until confluence. Two days after confluence (day 0), adipocyte differentiation was initiated with DMEM containing 10% FBS and a mixture of dexamethasone (1 μM), methylisobutylxanthin (0.5 mM), and insulin (1 μg/mL). On day 2, the medium was replaced by DMEM containing 10% FBS and insulin only. From day 4, cells were maintained in DMEM and 10% FBS, and the medium was refreshed every 2 days. Cells were treated with (0, 1, and 10 µM) flubendiamide, 5-aminoimidazole-4-carboxamide ribonucleotide (AICAR) (40 µM, dose was used as previously reported [13], or A769662 10 μM, used as previously reported [14]) from day 0 as indicated in each figure legend. We didn’t observe any influences of these concentrations of flubendiamide on cell viability measured by a previously reported 3-(4,5-dimethylthiazolyl-2)-2,5-diphenyltetrazolium bromide (MTT) based assay method [15] (Supplementary Figure S2).

### 2.3. Oil Red O Staining

Oil red O staining was performed as previously described with minor modification [16]. After washing with phosphate buffer saline (PBS), cells were fixed for 20 min with 10% neutral buffered formalin. Cells were then washed with sterile double distilled water and subsequently with 60% isopropanol for 2 min. Thereafter, the cells were stained with a filtered 0.35% Oil Red O solution dissolved in 60% isopropanol for 10 min and washed with PBS twice. Lipids were visualized by light phase contrast microscopy.

### 2.4. Triglyceride Quantification

Cells were washed twice with phosphate-buffered saline (PBS) and harvested by scraping in PBS containing 1% Triton-X after 8 days of differentiation. Homogenous samples were obtained from cells by sonication. The amount of triglyceride (TG) in the samples was measured with a commercial assay kit (Infinity™ Triglycerides Reagent; Thermo Scientific) and the protein content was measured with Bio-Rad DC protein assay kit following manufacturer’s instructions. The TG content was normalized with a protein concentration.

### 2.5. Immunoblotting

Cells were lysed with RIPA buffer containing a phosphatase inhibitor and a protease inhibitor cocktail. The protein concentrations were determined with the protein DC assay kit. Cell lysates containing 50 μg of protein were separated with 6% or 10% SDS-polyacrylamide gel and transferred to Immobilin P membrane (Millipore, Bedford, MA, USA). β-actin was utilized as an internal control. A horseradish peroxidase conjugated goat anti-rabbit IgG was utilized as the secondary antibody. Detections were made on an image Station Tanon 5500 (Shanghai, China) with ECL Substrate Kit (Bio-Rad Co., Hercules, CA, USA). Image and results were quantified with Image J software [17].

### 2.6. Statistical Analyses

Data were analyzed by the analysis of variance procedure (ANOVA) with the Statistical Analysis System (SAS Institute, Cary, NC, USA). Tukey’s multiple-range test was utilized to determine significant differences between groups. Significant differences were defined at the *p* < 0.05 level.

## 3. Results

### 3.1. Triglyceride Measurements in 3T3-L1 Adipocytes

**Figure 1:**
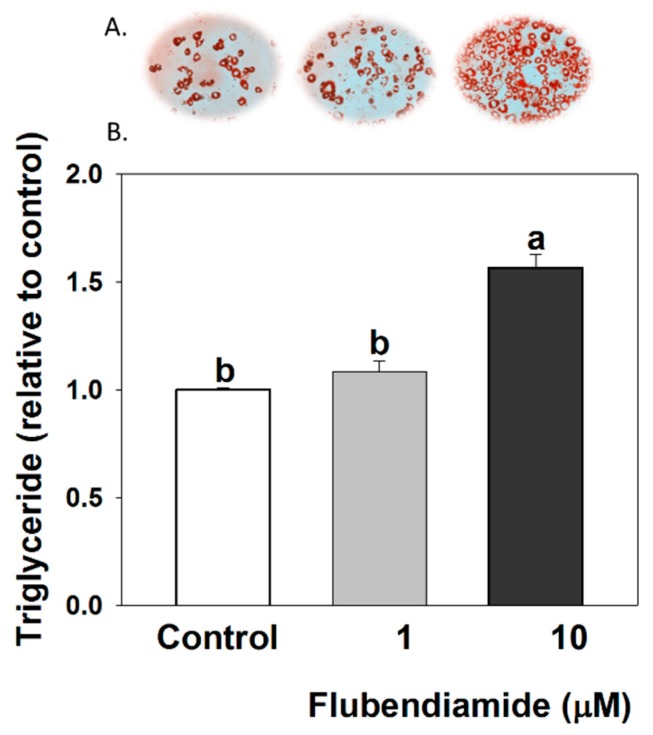
Flubendiamide treatment increased triglyceride accumulation in 3T3-L1 adipocytes. Cells were treated with flubendiamide for 8 days. (**A**) Oil red O staining; (**B**) triglyceride quantification with kit. Each value is expressed as the mean ± standard error of three separate experiments. Means with different letters were significantly different at *p* < 0.05 (a vs. b is significantly different).

Effect of flubendiamide on lipid accumulation in 3T3-L1 adipocytes is shown in Figure 1A (Oil red O representative picture) and Figure 1B (TG content measured by TG kit). Flubendiamide (10 µM) markedly increased TG content, compared to the control, but not at lower concentrations in the current model.

### 3.2. Influence of Flubendiamide on the Protein Expression of Regulators Controlling Adipocyte Differentiation and Lipid Metabolism

**Figure 2:**
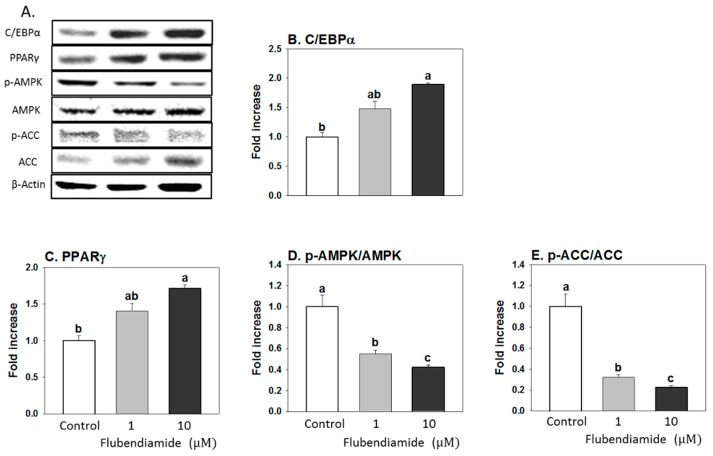
Effects of flubendiamide on protein levels of molecular mediators of adipogenesis. (**A**) representative picture; (**B**) C/EBP-α, CAATT element binding protein-α; (**C**) PPAR-γ, peroxisome proliferator-activated receptor-γ; (**D**) pAMPKα/AMPKα, AMP-activated protein kinase-α (inactive form)/phosphorylated AMPKα (active form), (**E**) pACC/ACC, acetyl-CoA carboxylase (active form)/phosphorylated ACC (inactive form). Cells were treated with flubendiamide for 8 days. Each value is expressed as the mean ± standard error of three separate experiments. Means with different letters were significantly different at *p* < 0.05 (a vs. b, a vs. c or b vs. c are significantly different, a vs. ab or ab vs. b are not significantly different).

Flubendiamide (10 µM) significantly increased expression of two key proteins regulating adipocyte differentiation, cytosine-cytosine-adenosine-adenosine-thymidine (CCAAT)/enhancer-binding protein α (C/EBPα) and peroxisome proliferator-activated receptor gamma (PPARγ), compared to the control (Figure 2B,C). AMPKα is a master regulator of energy production and lipid metabolism in the cell [18] and is activated by phosphorylation of threonine 172 (Thr172) [19,20]. Flubendiamide (10 µM) treatment significantly decreased the phosphorylation of AMPKα and its downstream target (Figure 2D,E). These results suggest that flubendiamide promotes adipocyte differentiation and lipogenesis, contributing to increased lipid accumulation at 10 µM.

### 3.3. Effect of AICAR on Adipogenesis Induced by Flubendiamide

**Figure 3:**
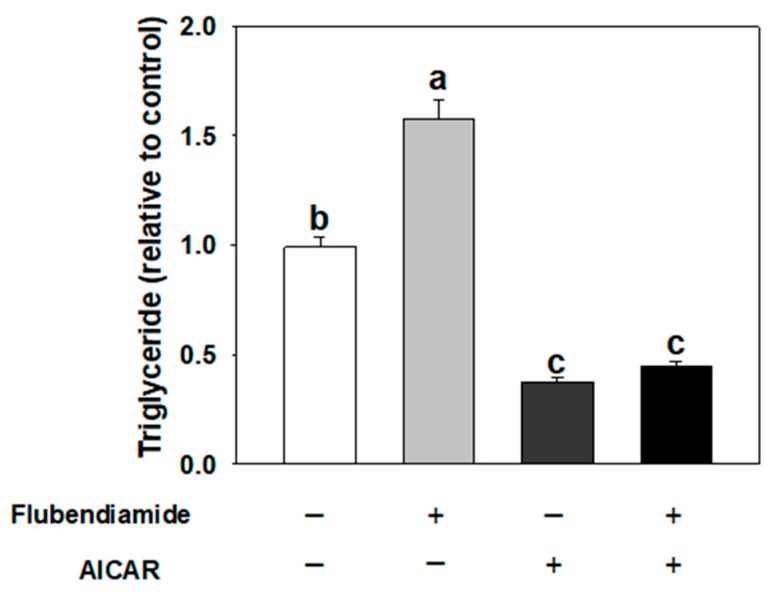
AICAR (5-Aminoimidazole-4-carboxamide ribonucleotide) abolished the increased fat accumulation induced by flubendiamide. Cells were treated with flubendiamide (10 μM) or AICAR (40 μM) for 8 days. Each value is expressed as the mean ± standard error of three separate experiments. Means with different letters were significantly different at *p* < 0.05 (a vs. b, a vs. c or b vs. c are significantly different).

We next determined whether AMPKα activation would inhibit enhanced adipogenesis induced by flubendiamide. 5-amino-4-imidazolecarboxamide riboside-1-β-d-ribofuranoside (AICAR), an adenosine analogue, is taken up by adenosine transporters on the cell membrane and then phosphorylated to generate 5-amino-4-imidazolecarboxamide ribotide (ZMP). ZMP mimics AMP and stimulates AMPK phosphorylation in the cell [21]. As shown in Figure 3, AICAR treatment alone inhibited fat accumulation, while 10 µM flubendiamide treatment alone enhanced fat accumulation. When cells were co-treated with AICAR and flubendiamide, the fat accumulation decreased, compared to flubendiamide treatment alone.

### 3.4. Influence of AICAR on Protein Expression of Regulators Controlling Adipogenesis

**Figure 4:**
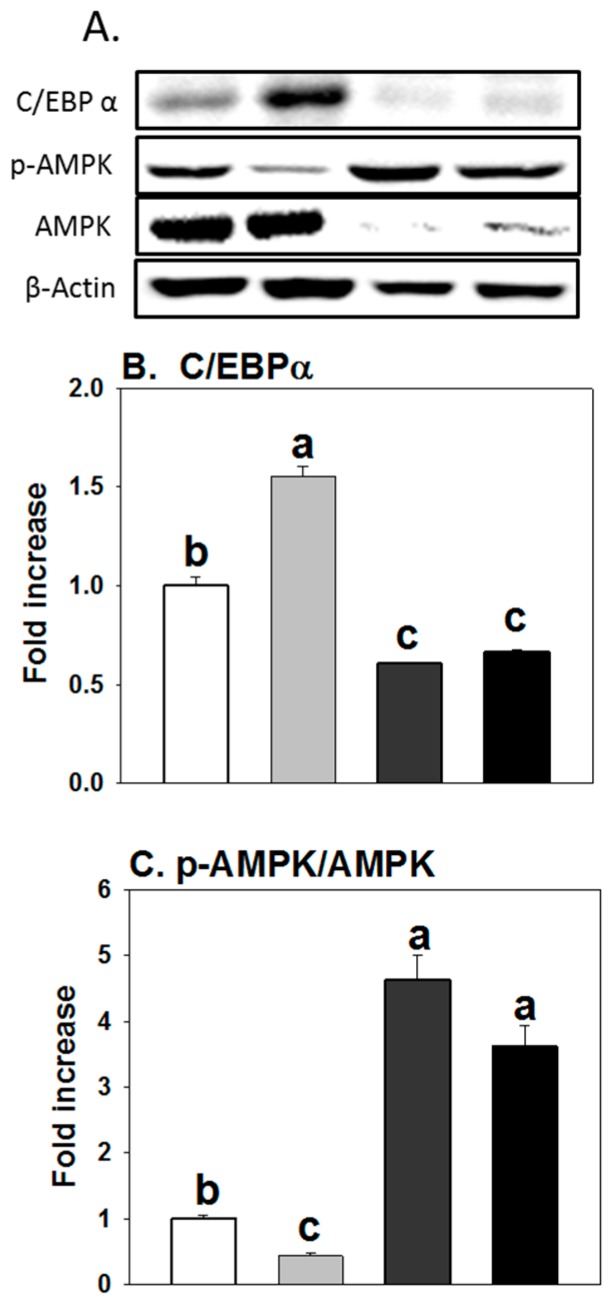
AICAR (5-Aminoimidazole-4-carboxamide ribonucleotide) abolished the increased expression of C/EBPα while flubendiamide induced the decreased expression of pAMPKα. A: representative picture; B: C/EBP-α, CAATT element binding protein-α; C: pAMPKα/AMPKα. Cells were treated with flubendiamide (10 μM) or AICAR (40 μM) for 8 days. Each value is expressed as the mean ± standard error of three separate experiments. Means with different letters were significantly different at *p* < 0.05 (a vs. b, a vs. c or b vs. c are significantly different).

As expected (Figure 4), AICAR treatment alone significantly decreased the expression of C/EBPα, while expressions of pAMPKα were significantly increased compared to the controls. When cells were treated together with flubendiamide and AICAR, increased protein expression of C/EBPα and decreased expression of pAMPKα induced by flubendiamide were abolished by AICAR treatment.

### 3.5. Influence of A769662 on Enhanced Adipogenesis Induced by Flubendiamide 

**Figure 5:**
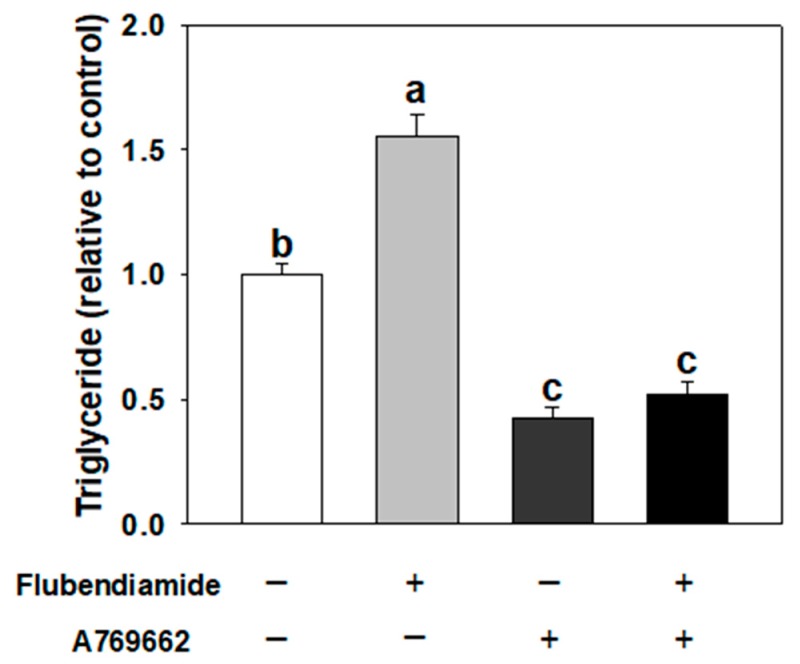
A769662 abolished the increased fat accumulation induced by flubendiamide. Cells were treated with flubendiamide (10 μM) or A769662 (10 μM) for 8 days. Each value is expressed as the mean ± standard error of three separate experiments. Means with different letters were significantly different at *p* < 0.05 (a vs. b, a vs. c or b vs. c are significantly different).

**Figure 6:**
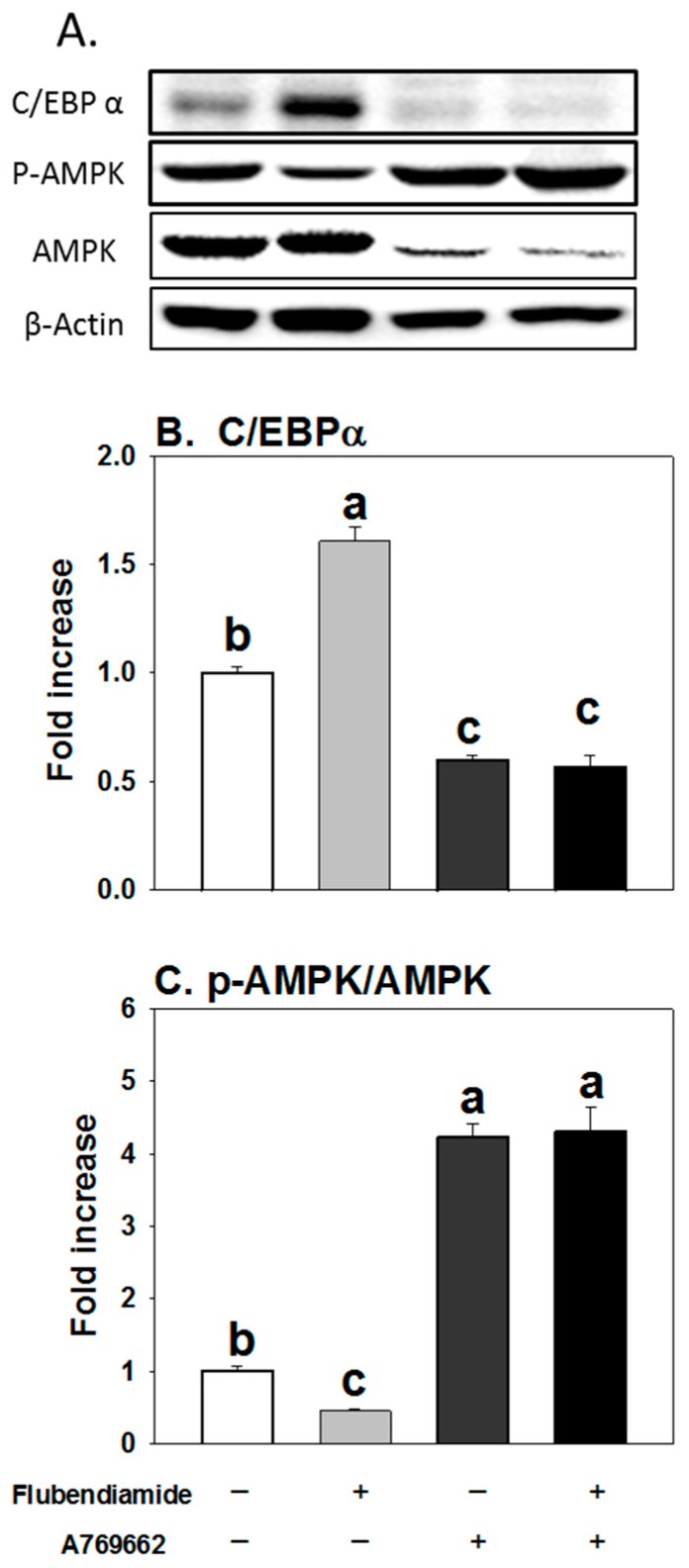
A769662 abolished the increased expression of C/EBPα and the decreased expression of pAMPKα induced by flubendiamide. (**A**) representative picture; (**B**) C/EBP-α, CAATT element binding protein-α; (**C**) pAMPKα/AMPKα. Cells were treated with flubendiamide (10 μM) or A769662 (10 μM) for 8 days. Each value is expressed as the mean ± standard error of three separate experiments. Means with different letters were significantly different at *p* < 0.05 (a vs. b, a vs. c or b vs. c are significantly different).

A769662, a potent activator of AMPK [14,22], was further utilized to investigate the effects of AMPK activation on increased adipogenesis by flubendiamide. As shown in Figure 5, when cells were co-treated with A769662 and flubendiamide, A769662 decreased the fat accumulation induced by flubendiamide, compared to the flubendiamide treatment alone. Similarly, increased protein expression of C/EBPα and decreased expression of pAMPKα induced by flubendiamide were abolished by A769662 (Figure 6A–C). These results suggest that AMPKα activation abolished enhanced adipogenesis induced by flubendiamide.

## 4. Discussion

The current results suggest that flubendiamide exposure resulted in enhanced adipogenesis in a 3T3-L1 cell model. To our knowledge, this is the first report linking enhanced adipogenesis to flubendiamide, particularly at 10 µM. Further study indicates that the AMPKα pathway is involved in increased adipogenesis triggered by flubendiamide.

AMPK is the downstream component of a protein kinase cascade that has a central role in maintaining energy balance and lipid metabolism [23]. Activation of AMPK has been reported to inhibit adipogenesis with reduced expression of PPARγ, C/EBPα and late adipogenic markers such as (fatty Acid Synthase) FAS and ACC [24]. Activation of AMPK has also been reported to inhibit lipogenesis by phosphorylation of ACC, the key regulated step in fatty acid synthesis and fatty acid oxidation. ACC catalyzes the synthesis of malonyl-CoA, a substrate of fatty acid synthesis, and is inhibited by AMPK-mediated phosphorylation of ACC [25]. The current results showed that AMPK activators (AICAR and A769662) subverted enhanced adipogenesis and increased expression of C/EBPα induced by flubendiamide. This suggests that flubendiamide may influence lipid metabolism via post-translational regulation of AMPK. With the current results, it is not clear if flubendiamide directly or indirectly targets AMPK. However, flubendiamide has previously been reported to increase intracellular Ca^2+^ levels, by activation of ryanodine-sensitive intracellular calcium release channels (ryanodine receptors) in insects [26]. Chronic elevation of intracellular calcium might inhibit the activation of AMPK, via calcium/calmodulin kinase kinase-β (CaMKKβ) [27,28]. There is a possibility that flubendiamide may influence intracellular calcium levels and result in altered adipogenesis and lipid metabolism via CaMKKβ-and AMPK- mediated mechanisms. However, flubendiamide’s effect on intracellular calcium needs to be further investigated in the future.

In the longer-term flubendiamide mice toxicology study, the toxicity symptoms of liver included organ weight increase, periportal fatty change, and hypertrophy, while kidney effects included increased kidney weight and nephropathy [29]. In *Helicoverpa armigera*, flubendiamide inhibited the larval growth, accelerated the Ca^2+^-ATPase activity and impeded mitochondrial function by interfering with complex I and F0F1-ATPase activity [30]. Flubendiamide was also reported to induce oxidative stress in *Daphnia magna* [31].

Currently, there is limited information concerning the serum levels of flubendiamide in humans. Levels of flubendiamide used in the present study might be higher than estimated exposure levels for most populations. However, flubendiamide might accumulate in adipose tissue or liver and reach relative high concentrations. For example, one previous publication reports that in female Fischer F344/DuCrj rats, which received oral doses of 200 mg/kg body weight daily for 1, 7 or 14 days, the highest plasma levels of flubendiamide reached around 20 μM (1.4 mg/L). Additionally, the level of flubendiamide in liver and fat reached as high as 19–27 mg/kg and 47–68 mg/kg, respectively. These results suggest that flubendiamide bioaccumulates in liver and adipose tissue, which might result in toxic effects of these tissues, such as fat accumulation, metabolic inflammation and insulin resistance [32].

**Figure 7:**
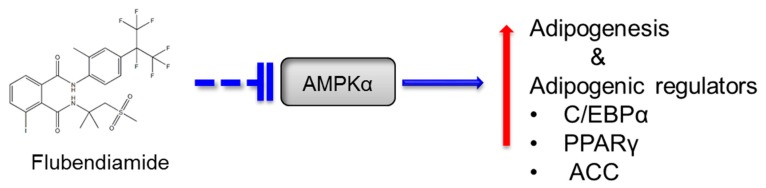
Flubendiamide promotes adipogenesis via inhibiting AMPKα. AMPKα, AMP-activated protein kinase-α; C/EBPα: CAATT element binding protein-α; PPAR-γ, peroxisome proliferator-activated receptor-γ; ACC, acetyl-CoA carboxylase.

To summarize, the present study suggests that flubendiamide enhanced adipocyte differentiation and increased fat accumulation in adipocytes (Figure 7) and is significant in providing a potential link between insecticide exposure, particularly flubendiamide, and adipogenesis. Nonetheless, the current results are limited to an in vitro model with relatively high flubendiamide doses. In addition, the role of flubendiamide metabolites needs to be studied. Thus, further in vivo and epidemiological studies of flubendiamide are necessary to further elucidate the significance of the current study.

Summary diagram:

## Supplementary Materials

The following are available online. Effects of flubendiamide treatment on triglyceride content (supplementary Figure S1) and cell viability (supplementary Figure S2) in 3T3-L1 cells.
