# Supplementary material for: Flubendiamide Enhances Adipogenesis and Inhibits AMPKα in 3T3-L1 Adipocytes
Source: Molecules. 2018 Nov 12;23(11):2950. doi: 10.3390/molecules23112950 (PMC6278525; doi:10.3390/molecules23112950)
Supplement: Supplementary file 1 [file molecules-23-02950-s001.pdf]

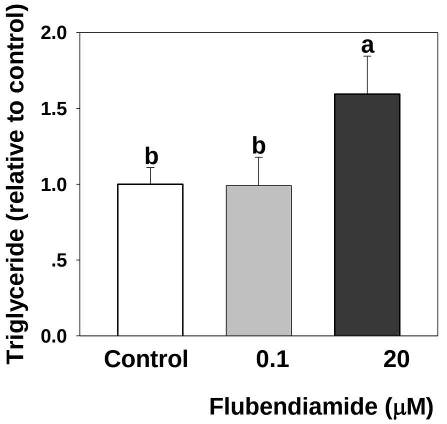

Supplementary figure 1. Effects of flubendiamide treatment on triglyceride content in 3T3-L1 adipocytes. Cells were treated with flubendiamide for 8 days. Each value is expressed as the mean ± standard error of three replicates.

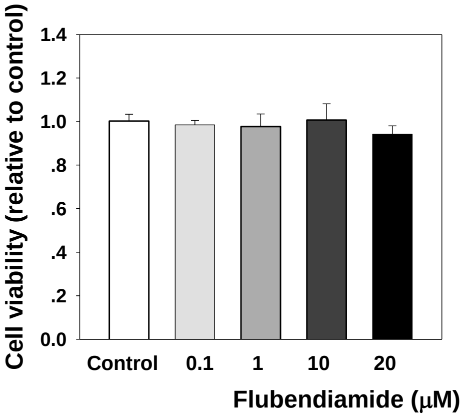

Supplementary figure 2. Effects of flubendiamide treatment on cell viability in 3T3-L1 adipocytes. Cells were treated with flubendiamide for 8 days. Each value is expressed as the mean ± standard error of six replicates.
